# *Clostridioides difficile* Infection in Children: A 5-Year Multicenter Retrospective Study

**DOI:** 10.3389/fped.2022.783098

**Published:** 2022-04-07

**Authors:** Danilo Buonsenso, Rosalia Graffeo, Davide Pata, Piero Valentini, Carla Palumbo, Luca Masucci, Antonio Ruggiero, Giorgio Attinà, Manuela Onori, Laura Lancella, Barbara Lucignano, Martina Di Giuseppe, Paola Bernaschi, Laura Cursi

**Affiliations:** ^1^Department of Woman and Child Health and Public Health, Fondazione Policlinico Universitario A. Gemelli, Rome, Italy; ^2^Dipartimento di Scienze Biotecnologiche di Base, Cliniche Intensivologiche e Perioperatorie, Università Cattolica del Sacro Cuore, Rome, Italy; ^3^Global Health Research Institute, Istituto di Igiene, Università Cattolica del Sacro Cuore, Rome, Italy; ^4^Dipartimento di Scienze di laboratorio e infettivologiche, Fondazione Policlinico Universitario A. Gemelli IRCCS, Rome, Italy; ^5^Unità di Oncologia Pediatrica, Fondazione Policlinico Universitario Gemelli IRCCS, Università Cattolica del Sacro Cuore, Rome, Italy; ^6^Internal Care Department, General Pediatric and Infectious Disease Unit, Bambino Gesù Children's Hospital, Rome, Italy; ^7^Department of Laboratories, Bambino Gesù Children's Hospital, Rome, Italy; ^8^Unit of Microbiology, Bambino Gesù Children's Hospital, Rome, Italy

**Keywords:** *C. difficile*, children, epidemiology CDI, risk factors CDI, prevention CDI

## Abstract

While there are numerous studies regarding *Clostridioides difficile* infection (CDI) in adults, literature on the pediatric population is scarce. Therefore, we performed a 5-year retrospective study between January 2014 and December 2018 in two referral centers in Rome, Italy. There were 359 patients tested for CDI who were enrolled: 87 resulted in positive and 272 in negative. CDI children had a higher number of previous-day hospital admissions (*p* = 0.024), hospitalizations (*p* = 0.001), and total hospital admissions (*p* = 0.008). Chronic comorbidities were more frequent in the CDI group (66.7% vs. 33.3%). Previous use of proton pump inhibitors and antibiotics was associated with CDI (*p* < 0.001). Among the antibiotics, only fluoroquinolones were significantly associated with CDI. Also, CDI children were more frequently exposed to antibiotics during the episode of hospitalization when children were tested. Our study provides an updated clinical and epidemiological analysis of children with CDI compared with a control group of children who tested negative. Further prospective studies could better define risk factors and preventive methods.

## Introduction

*Clostridioides difficile*, formerly known as *Clostridium difficile*, is a Gram-positive, spore-forming, anaerobic bacillus, which can exist both in toxigenic and non-toxigenic form (1). It is transmitted through the fecal–oral route or by direct contact. *C. difficile* is responsible for healthcare-associated diarrhea, but it is more common in adult with high mortality in elderly patients (2). Instead, *C. difficile* colonization is more frequent in the pediatric population, where most are asymptomatic. One major explanation is the absence of toxin-binding receptors in the immature intestinal mucosa of children (2). In recent years, the colonization rate has also increased in the pediatric population, even in the community setting (3, 4).

In specific conditions, *C. difficile* is able to colonize the large intestine, causing different consequences through the action of its toxins. In fact, *C. difficile* infection (CDI) can result in several conditions: from self-limiting secretory diarrhea to pseudomembranous colitis, toxic megacolon, and septic shock (5).

The development of CDI in children is the result of an altered balance in the host gut microbiota. This can be due to multiple causes, including the misuse of antibiotics, which is considered the most important risk factor for CDI (6). Moreover, *C. difficile* colonization may be promoted by other factors such as gastric acid suppression, gastrointestinal surgery, gastrostomy and jejunostomy tubes, and medications such as immunosuppressive drugs (7, 8).

Studies have shown that the epidemiology of this disease is changing (9). Consequently, we performed our study in order to define the clinical features and risk factors of CDI in children. Furthermore, the secondary aim was to evaluate the therapeutic regimens used and their impact on the outcome.

## Materials and Methods

We conducted a retrospective study on children younger than 18 years of age tested for CDI and admitted between January 2014 and December 2018 to the Department of Paediatrics of Policlinico Universitario Agostino Gemelli IRCSS and Ospedale Pediatrico Bambino Gesù IRCCS, two tertiary hospitals in the city of Rome, Italy.

Children with diarrhea (defined as the passage of at least three loose stools within a 24-h period); bloody diarrhea; other gastrointestinal, respiratory, or neurological symptoms; and risk factors (antibiotic and antacid therapy and prolonged hospitalization) were tested for CDI.

Given the high prevalence of asymptomatic colonization in literature, we did not routinely test for CDI among children less than 3 years old with diarrhea, unless specific risk factors (e.g., antibiotics, immunosuppression, and Hirschsprung) were present and alternative etiologies were excluded.

The laboratory diagnosis of CDI was based on the direct detection of *C. difficile* in a stool sample; adequate storage conditions are fundamental to prevent sample degradation and the relational false negative tests (10). This analysis occurred most commonly with an enzyme immunoassay (EIA), which provides a rapid turnaround time (about 1–2 h) as well as a sensitivity of 75–85% and a specificity of 95–100% (10). Due its low sensitivity, a test detecting *C. difficile* antigens is combined to EIA. This test is based on the detection of glutamate dehydrogenase (GDH). GDH is a metabolic enzyme that we detected with LIAISON® *C. difficile* GDH by DiaSorin.

**Figure 1:**
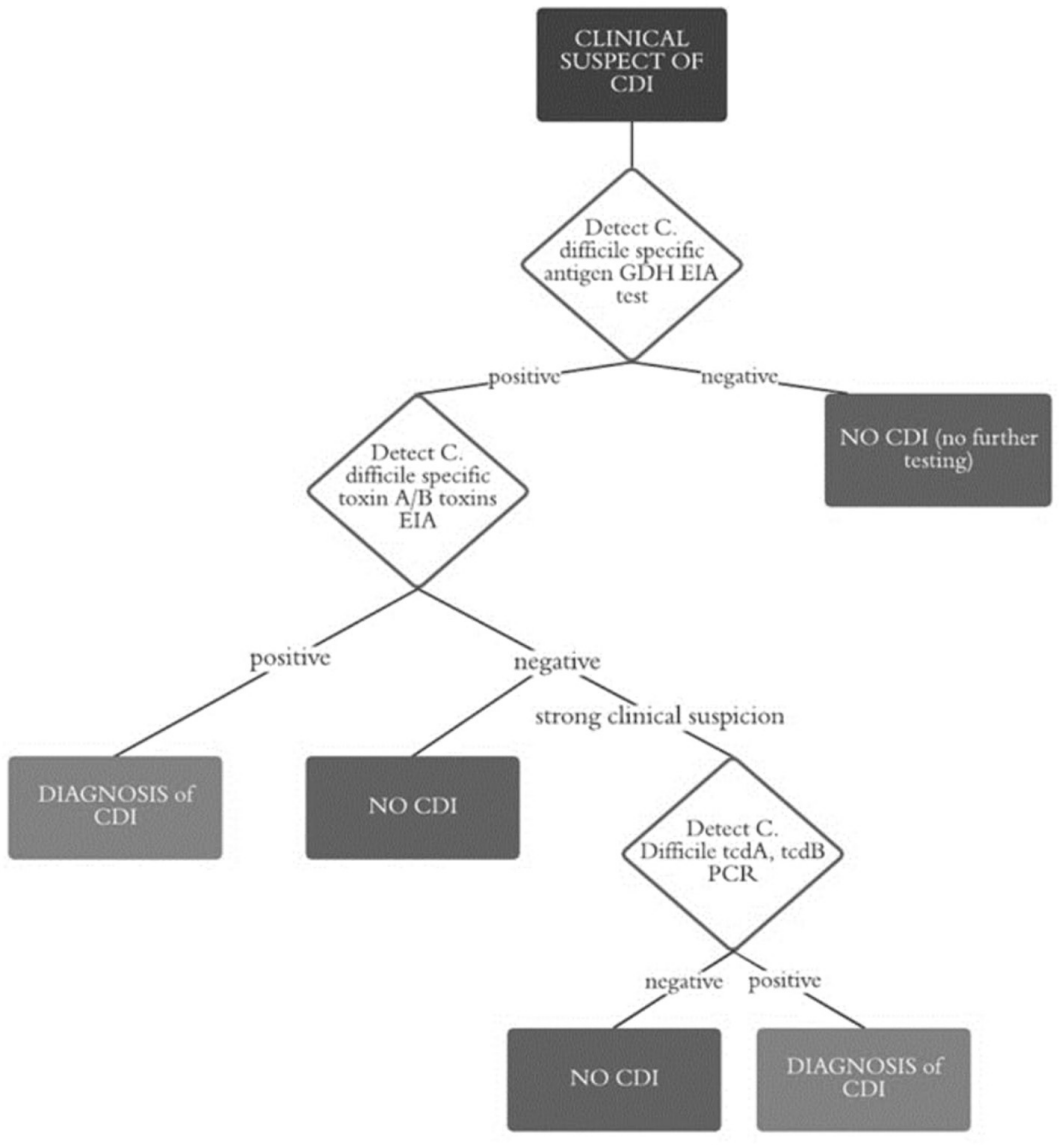
Flow chart diagnosis of CDI.

Since GDH is expressed by all strains of *C. difficile*, both toxigenic and non-toxigenic (it is also known as the common antigen), in order to confirm any toxin positivity, we carried out EIA for toxins A and B. In case of a negative EIA test but a strong clinical suspicion, Simplexa® *C. difficile* Direct Kit by DiaSorin (PCR real-time technology) for detection of toxins A and B genes was used (Figure 1).

Medical record information included demographics and laboratory data, cause and duration of hospitalization, antibiotic and antiacid therapy, CDI treatment, and comorbidities.

A descriptive statistical analysis was performed by constructing frequency tables (absolute and relative) for the categorical variables. Normality of continuous variables was checked using the Kolmogorov–Smirnov test. Between the two groups, categorical variables were tested using a chi-square test or Fisher's exact test, while continuous variables were analyzed with Student's *T* test (when data were normally distributed) or with the Wilcoxon test. *p*-values <0.05 were considered statistically significant. The analysis was performed with STATA v16.1.

The study was approved by the Ethics Committee of Fondazione Policlinico Universitario A. Gemelli IRCCS of Rome, Italy.

## Results

We enrolled 359 patients tested for CDI: 87 (24.2%) resulted in positive and 272 (75.8%) in negative. In the CDI-positive group, 85 were positive for GDH test and EIA, while another two were positive for RT-PCR (carried out despite the negativity of the former due to the strong clinical suspicion).

In the positive group (Table 1), the median age was 47 months: 53 (60.9%) were males, while 34 (39.1%) were females. Twenty-eight patients (32.2%) were admitted to the hospital with gastrointestinal symptoms (diarrhea, vomiting, hemorrhagic diarrhea, abdominal pain, dehydration, and eating difficulties) and 15 patients (17.2%) for follow-up, therapy, or surgery, while 19 patients (21.8%) presented other symptoms (cough, respiratory distress, rash, epileptic crisis, fever, edema, asthenia, hypotonia, and hematuria).

**Table 1:** Features of patients with CDI (*n* = 87).

Age in months (mean; SD)	47; 63.7
Male (*n*; %)	53; 60.9
**Reason for admission**	
Gastrointestinal (*n*; %)	28; 32.2
Treatment/surgery (*n*; %)	15; 17.2
Others (*n*; %)	19; 21.8
**Common symptoms**	
Fever (*n*; %)	22; 25.3
Diarrhea (*n*; %)	44; 50.6
**Inflammatory markers**	
C reactive protein (mean; SD)	16.4 mg/l; 74.5
Procalcitonin (mean; SD)	37.5 ng/ml; 52.9
**Comorbidities**	
Gastrointestinal (*n*; %)	11; 12.6
Cardiovascular (*n*; %)	10; 11.5
Neurological (*n*; %)	15; 17.2
Nephrological (*n*; %)	9; 10.3
Oncology (*n*; %)	10; 11.5
Genetics (*n*; %)	9; 10.3
None (*n*; %)	23; 26.4
**Previous hospitalization**	
Total number (mean; SD)	5.7; 7.2
Pediatrics (mean; SD)	15; 17.2
Oncology/hematology (mean; SD)	8; 9.2
NICU (mean; SD)	12; 13.8
PICU (mean; SD)	6; 6.9
**Treatment**	
Metronidazole (*n*; %)	27; 31.0
Vancomycin (*n*; %)	4; 4.6
Metronidazole and vancomycin (*n*; %)	12; 13.8
Fecal microbiota transplant (*n*; %)	1; 1.1
None (*n*; %)	7; 8.0

About common symptoms, 22 children (25.3%) presented fever, while diarrhea was found in 44 (50.6%). High levels of C-reactive protein (average 16.4 mg/l, SD 74.5) and procalcitonin (average 37.5 ng/ml, SD 52.9, but it was tested in a small number of cases) were found in this group. Among the 87 patients, comorbidities were generally present, and they were distributed as shown in Table 1.

Most of the positive patients had previous hospitalizations. The number of previous admissions averaged 5.7. The wards were different: 15 (17.2%) pediatrics, 8 (9.2%) oncology/hematology, 12 (13.8%) NICU, and 6 (6.9%) PICU.

Almost all children with CDI received treatment: 27 (31.0%) with mild/moderate symptoms were treated with metronidazole, while in 4 patients (4.6%), oral vancomycin was administered. Twelve of the children (13.8%) required both metronidazole and vancomycin; one patient (1.1%) was treated with fecal microbiota transplant due to his serious condition. Only seven children (8.0%) were not treated.

Table 2 reports the features of patients with CDI and age <3 years. The results are comparable, except for a higher percentage (44.1 vs. 32.2) of gastrointestinal pathologies as reason for hospitalization and symptoms such as fever (29.4 vs. 25.3) and diarrhea (55.9 vs. 50.6). On the other hand, in younger children, the inflammatory markers were lower (CRP 0.56 vs. 16.4), with also a lower number of comorbidities, oncological pathologies, and previous hospitalizations. Regarding the treatment, we reported a higher percentage of metronidazole (47.1 vs. 31.0) and no treatment (11.8 vs. 8.0) in this age group (Table 2).

**Table 2:** Features of patients with CDI and age <3 years (*n* = 34).

Age in months (mean; SD)	18.5; 9.6
Male (*n*; %)	22; 64.7
**Reason for admission**	
Gastrointestinal (*n*; %)	15; 44.1
Treatment/surgery (*n*; %)	3; 8.8
Others (*n*; %)	16; 47.1
**Common symptoms**	
Fever (*n*; %)	10; 29.4
Diarrhea (*n*; %)	19; 55.9
**Inflammatory markers**	
C reactive protein (mean; SD)	0.56 mg/l; 112.2
**Comorbidities**	
Gastrointestinal (*n*; %)	4; 11.8
Cardiovascular (*n*; %)	4; 11.8
Neurological (*n*; %)	5; 14.7
Nephrological (*n*; %)	3; 8.8
Oncology (*n*; %)	0
Genetics (*n*; %)	1; 2.9
None (*n*; %)	10; 29.4
**Previous hospitalization**	
Total number (mean; SD)	1.5; 2.1
Pediatrics (*n*)	7
Oncology/hematology (*n*)	1
NICU/PICU (*n*)	7
Surgery (*n*)	3
**Treatment**	
Metronidazole (*n*; %)	16; 47.1
Vancomycin (*n*; %)	0
Metronidazole and vancomycin (*n*; %)	4; 11.8
Fecal microbiota transplant (*n*; %)	0
None (*n*; %)	4; 11.8

### Comparisons Between the CDI-Positive and -Negative Groups

We compared the two groups, analyzing the clinical features and the risk factors they presented (Table 3).

**Table 3:** Comparison between CDI-positive and -negative groups.

	***C. difficile***	**No *C. difficile***	***P*-value**
Age in months (mean; SD)	47; 63.7	62.6; 69.8	0.194
Male (*n*; %)	53; 60.9	148; 54.4	0.468
**Ward**			
Pediatrics (*n*; %)	52; 60.3	133; 48.8	0.011
Oncology/hematology (*n*; %)	18; 20.5	29; 10.6	
NICU/PICU (*n*; %)	11; 12.3	82; 30.1	
Surgery (*n*; %)	6; 6.8	29; 10.6	
**Previous hospitalization**			
Previous DH admissions (*n*; SD)	6.9; 18.3	2.2; 5.2	0.024
Total number of previous hospital visits (*n*; SD)	13.8; 26.4	5.5; 8.0	0.008
Previous hospitalizations (*n*; SD)	5.7; 7.3	2.1; 3.2	0.001
**Comorbidities**			
Gastrointestinal (*n*; %)	11; 12.6	25; 9.3	<0.005 (Fisher's
Cardiovascular (*n*; %)	10; 11.5	5; 1.9	exact test)
Neurological (*n*; %)	15; 17.2	40; 14.8	
Nephrological (*n*; %)	9; 10.3	5; 1.9	
Oncology (*n*; %)	10; 11.5	0; 0.0	
Genetics (*n*; %)	9; 10.3	15; 5.6	
None (*n*; %)	29; 33.3	181; 66.7	
**Previous therapies**			
Proton pump inhibitors (*n*; %)	19; 21.8	13; 4.8	<0.001
Antibiotics (*n*; %)	33; 37.9	19; 7.0	<0.001
Penicillin (*n*; %)	11; 12.6	4; 1.5	0.185
Fluoroquinolones (*n*; %)	16; 18.4	2; 0.7	0.003 (Fisher)
Aminoglycosides (*n*; %)	5; 5.7	1; 0.4	0.397 (Fisher)
Cephalosporins (*n*; %)	12; 13.8	5; 1.8	0.291 (Fisher)
Carbapenems (*n*; %)	2; 2.3	0; 0.0	0.507 (Fisher)
Macrolides (*n*; %)	4; 4.6	3; 1.1	1 (Fisher)
Cotrimoxazole (*n*; %)	9; 10.3	6; 2.2	1 (Fisher)
Other antibiotics (*n*; %)	5; 5.7	3; 1.1	1 (Fisher)
**Therapies during hospitalization**			
Antibiotics (*n*; %)	56; 64.6	93; 34.2	0.005
Penicillin (*n*; %)	26; 30.4	103; 37.7	0.403
Fluoroquinolones (*n*; %)	16; 17.9	85; 31.1	0.09
Aminoglycosides (*n*; %)	24; 27.8	49; 18.0	0.212
Cephalosporins (*n*; %)	38; 43.6	80; 29.5	0.114
Carbapenems (*n*; %)	11; 12.6	5; 1.7	0.021
Macrolides (*n*; %)	9; 10.3	18; 6.6	0.422
Cotrimoxazole (*n*; %)	16; 17.9	0; 0.0	0.001

*SD, standard deviation; n, number; NICU, neonatal intensive care unit; PICU, pediatric intensive care unit; DH, day hospital*.

In the CDI-positive group, the mean age was 61.29 months and 53 were males, while in the CDI-negative group, the mean age was 62.6 and 148 were males.

The mean of previous-day hospital admissions was 6.9 (SD 18.3) in children with CDI and 2.2 (SD 5.17) in the negative group (*p*-value 0.024). The mean of previous hospitalizations was 5.7 (SD 7.28) in CDI positive and 2.1 (SD 3.17) in CDI negative (*p*-value 0.001), while the mean number of total hospital admissions was 13.8 (SD 26.4) and 5.54 (SD 7.95) in the positive and negative groups, respectively (*p*-value 0.008).

Both groups presented with chronic diseases, but these were more frequent in the positive group (66.7% vs. 33.3%). The most common chronic diseases were neurological diseases (17.2% for positive and 14.8% for negative) and gastrointestinal diseases (12.6 and 9.3%, respectively).

An important risk factor analyzed was the treatment with proton pump inhibitors (PPIs). It was found that PPIs were used by 21.8% of the positive group and by 4.8% of those who tested negative.

Antibiotic exposure is a known risk factor for CDI: our study showed a high percentage of patients treated with antimicrobial therapy before admission (in their entire life) in the positive group (37.9%), while only 7.0% of children received antibiotics in the negative group. The association was statistically significant (*p*-value <0.001).

Regarding the classes of previous antibiotics, in the positive group, most were fluoroquinolones (18.4%), followed by cephalosporins (13.8%) and penicillin (12.6%). Additional classes used were cotrimoxazole (10.3%), aminoglycosides (5.7%), carbapenems (2.3%), and others (5.7%). The negative group had a low percentage of antibiotic; in particular, 2.2% used cotrimoxazole, followed by cephalosporins (1.8%) and penicillin (1.5%). Fluoroquinolones, macrolides, and aminoglycosides were rarely administered. None of the CDI negatives were previously treated with carbapenems.

Antibiotic therapy during hospitalization was also evaluated as a possible risk factor. In the positive group, 64.6% of patients received antibiotics during hospitalization, while this percentage was lower in the negative group (34.2%, *p* = 0.005). Among those who received antibiotics, 30.4% of the CDI-positive and 37.7% of the CDI-negative patients used penicillin. Fluoroquinolones were administered in 17.9% of positive and 37.7% of negative patients. In the first group, 27.8% used aminoglycosides while 18% in the second. Cephalosporins were used by 43.6 and 29.5% of the positive and negative groups, respectively. Carbapenems were used by 12.5% of the positive group and only 1.7% of the negative group. Macrolides were used to treat 10.7% of children with CDI and 6.6% of negative patients. Cotrimoxazole was administered in 17.9% of positive patients, but it was not used in the negative group.

## Discussion

The aim of the study is to define the clinical features of CDI in children and the associated risk factors. While there are numerous data regarding *C. difficile* infection in adults, literature on the pediatric population is lower although increasing. Furthermore, some manuscripts suggest clinical/epidemiologic changes from past data (9).

Several significant aspects were found in our retrospective study.

Clinical manifestations (such as diarrhea and fever) alone do not help discriminate children with CDI. Most symptomatic patients had fever, watery or bloody diarrhea, and abdominal pain, but these symptoms were not more frequent in children of the positive group than in the negative group, as reported by Borali et al. (9).

Almost all children with CDI received treatment, most with metronidazole (31%), while only 8% of the patients were not treated. In accordance with previous studies (3–7), serious diseases are less common in pediatric age: 13.8% of patients required dual antibiotic therapy and one patient was treated with fecal microbiota transplantation due to CDI complications.

Among the risk factors analyzed, one of the most important is hospitalization. Indeed, the presence of frequent hospitalizations among children with *C. difficile* is statistically significant, as evidenced by other studies (4, 5). Our study reported more previous hospitalizations, total hospital admissions, and length of hospital stay in CDI-positive patients than in CDI-negative children. In fact, hospitalization could directly represent exposure to an environmental reservoir of *C. difficile* with its possible transmission and is indirectly associated with other risk factors such as the use of antibiotics and PPIs (11). In addition, we also reported a difference in the hospital wards. Patients with *C. difficile* infection were usually admitted to the pediatric ward. Positive patients were also mostly present in oncology, while the infection was more uncommon in PICU/NICU.

Comorbidities are another risk factor. They appear to be associated with CDI, and the most represented were neurological diseases and chronic gastrointestinal diseases (Table 2). The existence of underlying comorbidities is a feature already described among children with CDI (12). It is probably associated with factors such as hospitalization, the use of antibiotics or PPIs, and immunosuppression, all of which influence the composition of the normal intestinal microbial flora.

Our study reported a significant association between the onset of CDI and PPI use, according to the existing literature (13, 14). How PPI administration increases the risk of CDI is unclear. Gastric acidity is one of the defense mechanisms against bacterial infections of the gastrointestinal tract; therefore, its absence could support the transition from a spore to a vegetative form of *C. difficile* or, in any case, alter the normal balance of the intestinal microbial flora.

Antibiotic exposure is a known risk factor for CDI, especially after their discontinuation (3, 6, 7). We analyzed the use of antibiotics before and during hospitalization, and we found statistically significant differences between the two groups. Using a subgroup analysis, prior therapy with fluoroquinolones was associated with a higher risk of CDI, and this information is in agreement with other studies (15). Carrying out the same analysis regarding antibiotic therapy during hospitalization, treatment with carbapenems and cotrimoxazole was associated with CDI.

In two patients over 3 years of age, despite the negativity of the GDH test and EIA, due to the presence of prolonged and worsening diarrhea and risk factors (inflammatory bowel disease in the first and prolonged antibiotic therapy in the second), the diagnosis was made after the execution of NAAT.

Our study has several limitations. First, the retrospective investigation could bias the study results. Our investigation is based on secondary data from databases collected for clinical management and reused for research purposes; consequently, we are unable to obtain further information such as type of gastrointestinal comorbidities (for example IBD), outcome, and follow-up or state of immunosuppression. The small number of positive patients did not allow to analyze the differences and similarities between the various positive subgroups. Another limitation is the age of less than 3 years of some patients. In fact, although we tested the younger children only if symptomatic, with risk factors, and after exclusion of other causes, given the common colonization by *C. difficile* in this age group, the presence of a potential bias is possible. Finally, the execution of tests for CDI only in symptomatic or patients with risk factors did not allow us to calculate the prevalence of *C. difficile* colonization in our asymptomatic patients.

## Conclusions

We provided an updated clinical, epidemiological, and risk factor analyses of children with CDI compared with a control group of children that tested negative. Total hospitalizations, chronic comorbidities, and previous use of fluoroquinolones were significantly associated with CDI. Ulterior pediatric CDI studies are needed to confirm our findings. Further prospective studies could better define risk factors and the best preventive methods.

## Data Availability Statement

The original contributions presented in the study are included in the article/supplementary material, further inquiries can be directed to the corresponding author/s.

## Ethics Statement

The studies involving human participants were reviewed and approved by Ethic Committee of Fondazione Policlinico Universitario A. Gemelli IRCCS of Rome, Italy. Written informed consent to participate in this study was provided by the participants' legal guardian/next of kin.

## Author Contributions

DB, RG, and PV have given substantial contributions to the conception or the design of the manuscript. DP, CP, LM, AR, GA, MO, LL, BL, MG, PB, and LC have given substantial contributions to the acquisition, analysis, and interpretation of the data. All authors have participated to drafting the manuscript, and DB revised it critically. All authors contributed to the article and approved the submitted version.

## Conflict of Interest

The authors declare that the research was conducted in the absence of any commercial or financial relationships that could be construed as a potential conflict of interest.

## Publisher's Note

All claims expressed in this article are solely those of the authors and do not necessarily represent those of their affiliated organizations, or those of the publisher, the editors and the reviewers. Any product that may be evaluated in this article, or claim that may be made by its manufacturer, is not guaranteed or endorsed by the publisher.
